# Mealybugs nested endosymbiosis: going into the ‘matryoshka’ system in *Planococcus citri* in depth

**DOI:** 10.1186/1471-2180-13-74

**Published:** 2013-04-01

**Authors:** Sergio López-Madrigal, Amparo Latorre, Manuel Porcar, Andrés Moya, Rosario Gil

**Affiliations:** 1 Institut Cavanilles de Biodiversitat i Biologia Evolutiva, Universitat de València, Apartado Postal 22085, València, 46071, Spain; 2 Área de Genómica y Salud, Centro Superior de Investigación en Salud Pública (CSISP), Avenida de Cataluña 21, Valencia, 46020, Spain; 3 Fundació General de la Universitat de València, Apartado Postal 22085, València, 46071, Spain

**Keywords:** Nested endosymbiosis, *Planococcus citri*, *Moranella endobia*, *Tremblaya princeps*, functional complementation

## Abstract

**Background:**

In all branches of life there are plenty of symbiotic associations. Insects are particularly well suited to establishing intracellular symbiosis with bacteria, providing them with metabolic capabilities they lack. Essential primary endosymbionts can coexist with facultative secondary symbionts which can, eventually, establish metabolic complementation with the primary endosymbiont, becoming a co-primary. Usually, both endosymbionts maintain their cellular identity. An exception is the endosymbiosis found in mealybugs of the subfamily Pseudoccinae, such as *Planococcus citri*, with *Moranella endobia* located inside *Tremblaya princeps.*

**Results:**

We report the genome sequencing of *M. endobia* str. PCVAL and the comparative genomic analyses of the genomes of strains PCVAL and PCIT of both consortium partners*.* A comprehensive analysis of their functional capabilities and interactions reveals their functional coupling, with many cases of metabolic and informational complementation. Using comparative genomics, we confirm that both genomes have undergone a reductive evolution, although with some unusual genomic features as a consequence of coevolving in an exceptional compartmentalized organization.

**Conclusions:**

*M. endobia* seems to be responsible for the biosynthesis of most cellular components and energy provision, and controls most informational processes for the consortium, while *T. princeps* appears to be a mere factory for amino acid synthesis, and translating proteins, using the precursors provided by *M. endobia*. In this scenario, we propose that both entities should be considered part of a composite organism whose compartmentalized scheme (somehow) resembles a eukaryotic cell.

## Background

Symbiosis is a widespread natural phenomenon that has been postulated as one of the main sources of evolutionary innovation [1,2], and it is an example of compositional evolution involving the combination of systems of independent genetic material [3]. Many insects have established mutualistic symbiotic relationships, particularly with intracellular bacteria that inhabit specialized cells of the animal host (bacteriocytes). In most insect-bacteria endosymbioses described to date, host insects have unbalanced diets, poor in essential nutrients that are supplemented by their endosymbionts. Attending to their dispensability for host survival, we distinguish between primary (P) or obligate, and secondary (S) or facultative endosymbionts. P-endosymbionts are essential for host fitness and reproduction, and maternally transmitted through generations, while S-symbionts are not essential and can experience horizontal transfer. The genomes of P-endosymbionts usually exhibit an increase in their A + T content and undergo great size reduction, among other changes. The main evolutionary forces accounting for these features are relaxation of purifying selection on genes rendered unnecessary in the enriched intracellular environment, and random genetic drift due to a strong population bottlenecking throughout intergenerational transmission of the bacteria [4]. P and S symbionts can coexist in the same host. When an S-symbiont is also present, the irreversible genomic degenerative process could lead to the loss of some P-endosymbiont metabolic capabilities needed by the host. In this situation, two outcomes are possible: the host insect can recruit those functions from the S-symbiont, which then becomes a co-primary endosymbiont, establishing metabolic complementation with the former P-endosymbiont to fulfill the host needs or [5-8]; alternatively, the S-symbiont may replace its neighbor [9].

Mealybugs (Hemiptera: Sternorrhyncha: Pseudoccidae) form one of the largest families of scale insects, including many agricultural pest species that cause direct crops damage or vector plant diseases while feeding on sap [10]. All mealybug species analyzed so far possess P-endosymbionts. Two subfamilies have been identified, Phenacoccinae and Pseudococcinae [11], the latter having been studied in greater depth, all of which live in symbiosis with the β-proteobacterium “*Candidatus* Tremblaya princeps” (*T. princeps* from now on, for the sake of simplicity). Universal presence, along with the cocladogenesis of endosymbionts and host insects, led to *T. princeps* being considered the mealybug P-endosymbiont [12]. However, recently, other P-endosymbionts from the β-proteobacteria and Bacteroidetes groups have been identified in the subfamily Phenacoccinae [13]. Most genera of the subfamily Pseudococcinae also harbor additional γ-proteobacteria endosymbionts that, due to their discontinuous presence and polyphyletic origin, have been considered as S-symbionts [14]. An unprecedented structural organization of the endosymbionts of the citrus mealybug *Planococcus citri* was revealed by von Dohlen and coworkers [15]: each *T. princeps* cell harbors several S-endosymbiont cells, being the first known case of prokaryote-prokaryote endocelullar symbiosis. The S-endosymbiont has recently been named “*Candidatus* Moranella endobia” (*M. endobia* from now on) [16]. The dynamics of both endosymbiont populations throughout the insect life-cycle and their differential behavior depending on host sex [17] suggest that both play an important role in their hosts’ nutritional and reproductive physiology, putting into question the secondary role of *M. endobia.*

The sequencing of two fragments of the genome of *T. princeps* from the pineapple mealybug, *Dysmicoccus brevipes*[18], showed a set of unexpected genomic features compared with that found in most P-endosymbiont reduced genomes. This species presents a rather high genomic G + C content – a rare condition among P-endosymbionts with the only known exception being “*Candidatus* Hodgkinia cicadicola” (P-endosymbiont of the cicada *Diceroprocta semicincta*[7]) –, a partial genomic duplication including the ribosomal operon and neighbor genes, and low gene density. All other sequenced genomes from endosymbionts having a long relationship with their host maintain a single set of rRNA genes, therefore these data suggested an unprecedented complexity for this P-endosymbiont genome, an unexpected finding for a long co-evolutionary process, as already elucidated for this symbiotic system [18]. However, the recent sequencing of two strains of *T. princeps* from *P. citri* (PCIT and PCVAL) has shown that it is, in fact, the smallest (139 kb) and most simplified bacterial genome described to date [16,19]. Functional analysis reveals that the genetic repertoire of *T. princeps* is unable to sustain cellular life, according to Gil et al. (2004) [20], and that it entirely depends on *M. endobia* for many essential functions. Even though most of its genome is occupied by ribosomal genes and genes involved in the biosynthesis of essential amino acids, *T. princeps* likely depends on its symbiotic consortium partner to build its own ribosomes and for amino acid production [16,19].

The work published by McCutcheon and von Dohlen [16] mainly focused on the analysis of the *T. princeps* genome and detangling the amino acid biosynthetic pathways in which all three partners (*T. princeps*, *M. endobia* and the host) appear to be involved. However, the characteristics and functionality of the *M. endobia* genome, as well as other possible modes of complementation between the two endosymbionts, have remained largely unexplored. In this work we present a comprehensive analysis of the predicted consortium functional capabilities and interactions, thus offering new insights into how this bacterial consortium may function internally. Additionally, we have performed a comparative analysis of both endosymbiont genomes in two *P. citri* strains, PCIT [16] and PCVAL ([19] and this work). Our analysis suggests that both genomes have undergone reductive evolution, albeit with some unusual genomic features, probably as a consequence of their unprecedented compartmentalized organization.

## Results and discussion

### Main features and genomic variability between two strains of *P. citri* nested endosymbionts

The main molecular features of the genomes of *T. princeps* str. PCVAL [19] and PCIT [16], and *M. endobia* str. PCVAL (this work) and PCIT [16] are summarized in Table 1. It is worth mentioning that differences in CDS numbers and coding density between both strains are due to differences in the annotation criteria used, since the number of polymorphisms detected between the two sequenced strains of *T. princeps* and *M. endobia* is minimal (see Additional file 1 for a list of annotation differences in CDS and tRNA genes).

**Table 1:** **Main genomic features of the two strains of the *P. citri *endosymbiotic consortium already sequenced**

	***T. princeps *PCVAL**	***T. princeps *PCIT**	***M. endobia *PCVAL**	***M. endobia *PCIT**
GenBank accession number	CP002918	CP002244	CP003881	CP002243
Genome size (bp)	138931	138927	538203	538294
Total gene number	130	136	458	452
CDSs	116	121	411	406
rRNAs	6	6	5	5
tRNAs	7	8	41	41
Small RNA genes	1	1	1	0
Pseudogenes	19 (CDS) 6 (tRNA)	19 (CDS) 4 (tRNA)	25 (CDS)	23 (CDS)
Overall gene density (%)	71.2	72.9	79.3	79.0
Average ORF length (bp)	775	760	1012	1022
Average IGRs (bp)	466.8	389.0	260.3	268.0
G + C content (%)	59.0	58.8	44.0	43.5
genes	58.6	58.5	45.5	45.4
pseudogenes	58.8	59.9	43.6	44.7
IGR	59.4	59.5	36.0	36.2

Data referring to strain PCIT have been obtained from the GenBank database.

Both consortium partners lack a canonical *ori*C, which is consistent with the absence of *dnaA,* similarly to many other reduced endosymbiont genomes already sequenced (e.g., *Blochmannia floridanus*[21], *Wigglesworthia glossinidia*[22], *Carsonella rudii*[23], *Hodgkinia cidadicola*[24], *Zinderia insecticola*[8], and *Sulcia muelleri*[25]). This has been considered an indication that the endosymbionts rely on their host for the control of their own replication [21]. Another shared genomic characteristic of both endosymbionts is their low gene density (already noticed in [16] for *T. princeps*) and the large average length of the intergenic regions, in which no traces of homology with coding regions of other bacteria can be found. Although these traits are unusual in bacterial endosymbionts, they have also been described for *Serratia symbiotica* SCc, the co-primary endosymbiont of *Buchnera aphidicola* in the aphid *Cinara cedri*[5]. This non-coding DNA is probably the remnant of ancient pseudogenes that are gradually being eroded [26].

**Figure 1:**
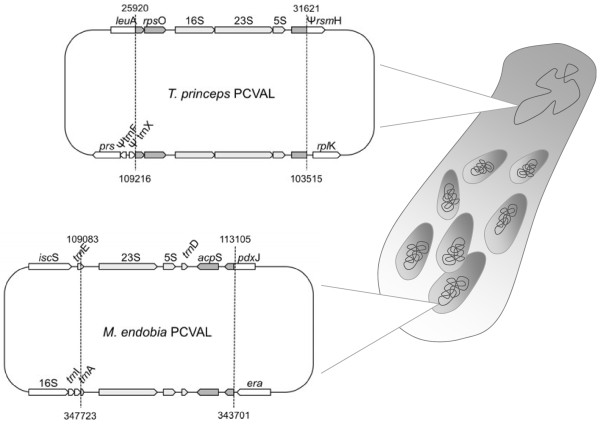
**Endosymbionts partial genome duplications.** Duplicated regions evolving under concerted evolution in *T. princeps* and *M. endobia* are represented. Only affected genes (grey arrows: coding genes; light grey arrows: RNA genes) and their closest neighbors (white arrows) are depicted. Numbers indicate the location of these duplicated regions in the corresponding genomes.

Another remarkable feature, compared with other endosymbiotic systems, is that both *T. princeps* and *M. endobia* display one partial genomic duplication event involving the ribosomal operon (Figure 1). The duplication in *T. princeps* has been described in other mealybugs [18], and it affects the rRNA genes (*rrsA, rrlA* and *rrfA*) plus *rpsO* (encoding ribosomal protein S15). Ribosomal genes and *loci* from its closest genomic context (*acpS* and partial *pdxJ*) are also duplicated in *M. endobia* but*,* unlike in *T. princeps*, the two copies of the *M. endobia* ribosomal operon have not remained intact. Comparative synteny among several γ-proteobacteria species suggests that the additional copy was inserted in the lagging strand, while the original copy suffered the losses. Thus, although 4 kb of the duplicated region (positions 109,083-113,105 and 343,701-347,723 for the copies in the direct and lagging strand, respectively) seem to be under concerted evolution (both regions are identical in both genomes), the original copies of *rrsA, trnI* and *trnA* have been lost.

The reductive process affecting both genomes has led to the loss of most regulatory functions. Thus, they lack most regulatory genes and some genes have lost regulatory domains. This is the case of *met*L and *adk* from *T. princeps*, which have lost the regulatory ‘ATC’ domain, or the loss of the ‘HTH’ domain of *bir*A, the ‘PNPase C’ domain of *rne* and the ‘DEAD box A’ of *dead* in the case of *M. endobia.* Additionally, many other genes have been shortened due to frameshifts or the presence of premature stop codons, in comparison with their orthologs in free-living relatives (e.g. *ssp*B, *rpl*Q, *rpl*O and *aro*C in *T. princeps; thi*C, *ybg*I, *yac*G, *ygb*Q, *fts*L, *fts*Y and *til*S in *M. endobia*). In some cases, the shortening removes some non-essential protein domains completely (e.g., *eng*A, *rpo*A and *rpo*D in *T. princeps; sec*A, *ace*F, *yeb*A and *met*G in *M. endobia*). The loss of the ‘anticodon binding domain of tRNA’ and ‘putative tRNA binding domain’ of *met*G, encoding methionyl-tRNA synthetase is common to other endosymbionts with reduced genomes.

**Figure 2:**
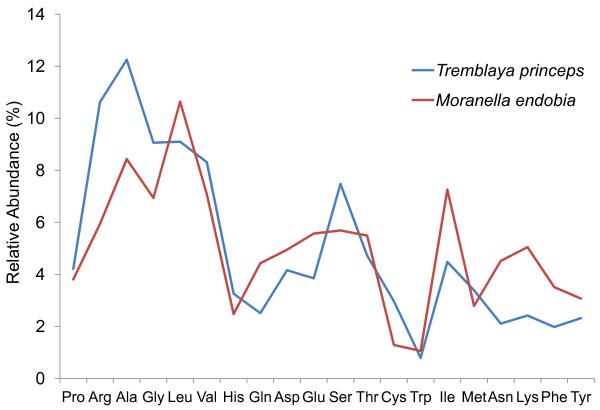
**Amino acid content profiles for *T. princeps *and *M. endobia *proteomes.** Amino acids are ranked from left to right according to the GC-richness of the corresponding codons (see Additional data file 2).

Finally, even though both genomes have an unusually high G + C content compared with most bacterial endosymbionts, at least *M. endobia* seems to be suffering the AT mutational bias typical of bacterial genomes [27,28]. This conclusion is drawn from the analysis of the nucleotide composition of genes, pseudogenes and IGRs (Table 1), as well as the preferential use of AT-rich codons (Additional file 2) including a high incidence of the TAA stop codon (56.44%). Since both genomes seem to rely on the DNA replication and repair machinery of *M. endobia* (see next section), both genomes could be expected to undergo a similar trend towards an increase in AT content. However, this trend is undetectable in *T. princeps*, where the G + C content of pseudogenes and IGRs do not differ from that of the genes (Table 1). The differences in G + C content between both genomes could be due to a higher ancestral G + C content plus a slower evolutionary rate for *T. princeps*, due to its extreme genome reduction, and the biology of the system (i.e., a lower replication rate, since each *T. princeps* cell retains several *M. endobia* cells). In fact, the codon usage bias (Additional file 2) and differences in the amino acidic composition between both endosymbiont proteomes (Figure 2) reflect their differences in G + C content. Thus, *T. princeps* proteins are rich in amino acids encoded by GC-rich codons (Ala, Arg, Leu, Gly, Val and Ser represent 56.82% of the total, whereas Phe and Trp are scarce), while *M. endobia* has a weaker amino acid composition bias (Additional file 2).

#### T. princeps genome comparison

The genome alignment of both *T. princeps* strains showed a high degree of identity at the sequence level (99.98%, being 138,903 bp identical), which is coherent with their evolutionary proximity and extreme genome reduction. Although we also detected the 7,032-bp region flanked by 71-bp inverted repeats described in the strain PCIT [16], we only found one orientation in the population used for genome sequencing.

The genome of strain PCVAL only differs in 4 nucleotides in length from strain PCIT [16], involving five short indel events of one (4 cases) or two nucleotides (1 case). Additionally, 23 nucleotide substitutions were detected. Transitions represent 43.5% (10/23) of the total substitutions. Although the number of mutations is too small to be representative and, therefore, it is difficult to draw clear conclusions, it is noteworthy that all indels plus 87% of the detected substitutions between both strains are located in the coding fraction of the genome, in spite of its low coding density. One of the detected indels affects the start codon of *aroC*, involved in the biosynthetic pathway of aromatic amino acids, which is then changed to a GTG start codon. Two other short deletions yield the loss (AT) and recovery (T) of the reading frame of *ilvD*, needed for the synthesis of isoleucine and valine. The non-inactivating character of these mutations on genes involved in biosynthetic pathways of essential amino acids without an ortholog in the genome of *M. endobia*, corroborates their importance for the bacterial partnership. The other two indels, as well as 20 out of 23 of the observed substitutions, were located at the 3^′^ end of *rplQ*, which suggests that this region could be a mutational hot-spot. To confirm this point, we analyzed the original *P. citri* DNA samples used in the genome sequencing experiments by PCR amplification of the *rplQ* and flanking ITS regions, as well as new DNA samples obtained from individual insects cultivated in Almassora (Spain) and from environmental colonies collected in Murcia (Spain). Although all three samples were obtained from different plant hosts and separated by more than 300 Km, they were identical. Since we have no direct availability of the PCIT strain, it is feasible that the Spanish and American populations differ.

#### M. endobia genomes comparison

The alignment of both genomes of *M. endobia* showed that the genome of strain PCVAL is 65 nucleotides shorter than that of PCIT, and allowed the identification of 262 substitutions. Among them, 90.1% were G/C↔A/T changes, with only 18 A↔T changes and 8 G↔C changes, which is additional indirect evidence of the mutational bias towards A/T already observed in the codon usage analysis (Additional file 2). As expected for a neutral process, the mutational bias affected both strains equally, being the changes G/C↔A/T evenly distributed (50.4% A/T in strain PCIT and 49.5% in PCVAL). Regarding the genome distribution of the polymorphisms, 47% of them (123) map onto IGRs, and 4.5% (12) onto 10 pseudogenes. The 139 substitutions detected in the coding fraction affect only 111 out of the 406 orthologous genes. Among these substitutions, 77 are synonymous (dS = 0.0011 ± 0,0001), and 62 non-synonymous (dN = 0.0005 ± 0,0000), with a ω = 0.44, suggesting the action of purifying selection. It is worth noticing that about 75% of them affect functional domains, suggesting that many putatively functional genes accumulate mutations, which also justifies the maintenance of a minimal set of molecular chaperones to help in the proper folding of the encoded proteins.

Additionally, 60 indels were detected between both *M. endobia* strains, with a mean size of 5.4 nucleotides, although there is a great variance, between 1 and 75 nucleotides. Results showed 58.3% (35/60) of the indels affect homopolymers of A (22/39), T (12/36) and, less frequently, G (5/37) and C (3/35), which is consistent with the higher proportion of A and T homopolymers. This fact may be related with the above-mentioned A/T mutational bias. Although artifacts due to sequencing errors cannot be ruled out, given that PCVAL genomes were assembled based on 454 sequencing data, there are several pieces of evidence that indicate that the observed indels may be real. First, although homopolymers can be found both in coding and non-coding regions, most indels affect the non-coding parts of the genome. Second, even when A/T homopolymers are quite abundant in the *M. endobia* genome (844 cases equal to or bigger than 6 nucleotides), only a small fraction of them are affected by indels (29 cases, representing 3.4%). Finally, the coverage of the affected regions was always higher than 27X, and the PCVAL reads polymorphism was almost null. The remaining indels affect microsatellites of 2 to 8 nucleotides with a small number of copies. Forty-seven indels (78.3%) map onto intergenic regions, pseudogenes (2 in Ψ*pdxB*, 1 in Ψ*prfC*) or the non-functional part of shortened genes (*dnaX*), and only 13 indels (21.7%) map onto coding regions. Most of these are located on the 3^′^ end of the affected gene, causing enlargement or shortening of the ORFs compared with the orthologous gene in other γ-proteobacteria. Thus, *glyQ* (involved in translation) and *ptsI* (participating in the incorporation of sugars to the intermediary metabolism) are enlarged in strain PCVAL, while *rpp*H (involved in RNA catabolism) is shortened in this strain without affecting described functional domains. Conversely, the shortening of *fis* (encoding a bacterial regulatory protein) in PCVAL, and of *yicC* (unknown function) and *panC* (involved in the metabolism of cofactors and vitamins, a function that is incomplete in *M. endobia*) in PCIT, affect some functional domains, although their activity might not be compromised. Finally, amino acid losses without frameshift were observed in PCVAL (relative to PCIT) for the *loci holC* (encoding subunit chi of DNA polymerase III), *rluB* (involved in ribosome maturation), *surA* (encoding a chaperone involved in proper folding of external membrane proteins), and *pitA* (encoding an inorganic phosphate transporter). None of the corresponding functional domains were affected in the first two cases, while the indel polymorphisms mapped inside the ‘PPIC-type PPIASE’ domain in *surA*, which appears to be dispensable for the chaperone qualities of the protein [29]*.* Therefore, it seems that most (if not all) changes that could affect the functions of the encoded proteins have been removed by the action of purifying selection.

### Functional analysis of the nested consortium

Most endosymbiotic systems analyzed to date at the genomic level have a nutritional basis, and many of them involve the biosynthesis of essential amino acids that are in short supply in the host diet. The metabolic pathways leading to amino acid biosynthesis in the *T. princeps*-*M. endobia* consortium found in *P. citri* were recently analyzed in detail by McCutcheon and von Dohlen [16] and, therefore, they will not be dealt with in this study. These authors also stated that *T. princeps* is unable to perform DNA replication, recombination or repair by itself, and the same applies to translation. They speculate that a passive mechanism such as cell lysis could provide *T. princeps* with the needed gene products from *M. endobia.* Our present work provides a detailed analysis of the *M. endobia* functional capabilities, based on a functional analysis of its genome, regarding informational functions or other intermediate metabolism pathways beyond amino acids biosynthesis. In the following sections these functional capabilities will be analyzed in a comprehensive manner, considering both endosymbiotic partners, in order to identify putative additional levels of complementation between them.

#### DNA repair and recombination

Contrary to what is found in bacterial endosymbionts with similarly reduced genomes, *M. endobia* has quite a complete set of genes for DNA repair and recombination, while none were annotated in the *T. princeps* genome [16,19]. Although it has lost the nucleotide excision repair genes (only *uvrD* is present), *M. endobia* retains a base excision repair system (the DNA glycosylases encoded by *mutM* and *ung* plus *xth,* the gene encoding exonuclease III, involved in the repair of sites where damaged bases have been removed). The mismatch repair system is also almost complete, since only *mutH*, encoding the endonuclease needed in this process to cleave the unmethylated strand, has been lost. Additionally, *M. endobia* also retains almost the entire molecular machinery for homologous recombination (*rec*ABCGJ, ruvABC, priAB), which could be responsible for the concerted evolution of the duplications in both genomes. In the absence of *rec*D, the RecBC enzyme can still promote recombination, since it retains helicase and RecA loading activity. The missing exonuclease V activity can be replaced by other exonucleases with ssDNA degradation activity in the 5^′^ → 3^′^ sense, such of RecJ [30], which has been preserved. The final step in homologous recombination requires the reloading of origin-independent replication machinery. Two replisome reloading systems have been described in *E. coli*, one of which requires the participation of PriA, PriB and DnaT [31], and it appears that helicase DnaB loading and unwinding of a replication fork is dependent upon the activities of DnaT and DnaC, among other restart proteins. These last two proteins are the only two elements of the replisome that are not encoded in the *M. endobia* genome. However, mutations in *dna*C which have the ability to bypass such requirements in the loading of DnaB have been described [32], and *dna*C is also absent in other reduced genomes that have been characterized (e.g. *Blochmannia floridanus*[21], *Wigglesworthia glossinidia*[22] or *Mycoplasma genitalium*[33]). Additionally, the role of DnaT in primosome assembly has not been fully elucidated [34]. Therefore, it cannot be ruled out that *dna*T is not essential for the functioning of the homologous recombination system in this bacterial consortium.

#### RNA Metabolism

Even though most genes present in the *T. princeps* genome are involved in RNA metabolism (78 out of 116 genes, occupying 35% of its genome length and 49% of its coding capacity) [16,19], it still seems to depend on *M. endobia* for transcription and translation. Thus, *T. princeps* encodes every essential subunit of the core RNA polymerase (*rpo*BCA) and a single sigma factor (*rpo*D), but no other genes involved in the basic transcription machinery or in RNA processing and degradation are present in its genome. On the other hand, *M. endobia* possesses a minimal but yet complete transcription machinery [35] plus some additional genes, including the ones encoding the ω subunit of the RNA polymerase (*rpo*Z), the sigma-32 factor (*rpo*H), and the transcription factor Rho. It also presents several genes involved in the processing and degradation of functional RNAs, i.e. *pnp*, *rnc* (processing of rRNA and regulatory antisense RNAs), *hfq* (RNA chaperone), *rne*, *orn*, *rnr* (rRNA maturation and mRNA regulation in stationary phase), and *rpp*H (mRNA degradation). It is surprising that the small genome of *M. endobia* has also retained several transcriptional regulators, the functions of which are not yet fully understood, and which are absent in other endosymbionts with reduced genomes. These include CspB and CspC (predicted DNA-binding transcriptional regulators under stress conditions), and NusB, which is required in *E. coli* for proper transcription of rRNA genes, avoiding premature termination [36]. *cpx*R, encoding the cytoplasmic response regulator of the two-component signal transduction system Cpx, the stress response system that mediates adaptation to envelope protein misfolding [37], is also preserved, while the companion sensor kinase *cpx*A appears to be a pseudogene. This might be an indication of a constitutive activation of the regulatory protein.

**Figure 3:**
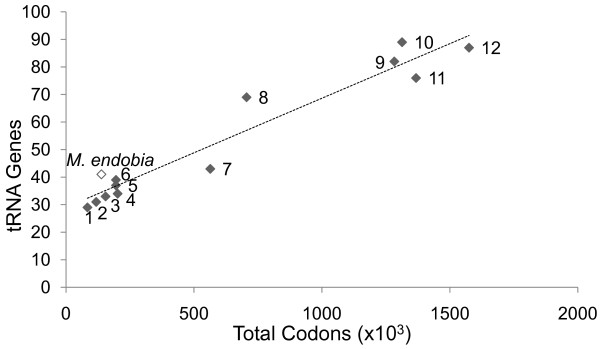
**Correlation between tRNA genes content and translational requirements.** Selected genomes with variable translational requirements are taken into account: *Sulcia muelleri* CARI (1)*, Buchnera aphidicola B*Cc (2), *Moranella endobia* PCVAL (white), *Riesia pediculicola* (3), *Blatabacterium* sp. Bge (4), *Blochmania floridanus* (5), *Baumania cicadicolla* (6), *Hamiltonella defensa* (7), *Sodalis glossinidius* (8), *Yersinia enterocolitica* subsp. Enterocolitica 8081 (9), *Escherichia coli* str. K-12 MG1655 (10), *Dickeya dadantii* Ech586 (11), and *Serratia* sp. AS9 (12). A high correlation between both parameters was observed when every genome except *M. endobia* were included (R^2^ = 0.94), as well as when only endosymbionts except *M. endobia* were considered (R^2^ = 0.77). Inclusion of *M. endobia* among endosymbionts caused a drastic diminution of the coefficient (R^2^ = 0.33).

Regarding translation, an extremely complex case of putative complementation between both bacteria is predicted, which would represent the first case ever described for this function. Thus, only *M. endobia* presents the genes *fmt* and *def*, responsible for the synthesis of formil-methionil-tRNA and methionine deformilation, respectively, and a minimal set of genes for tRNA maturation and modification [35], as well as a complete set of aminoacyl-tRNA synthetases. Additionally, it codes for more than 80% of the tRNA genes annotated in both genomes and, therefore, is supposed to be the source of these tRNAs for the whole consortium. Comparative analysis with other endosymbiotic or free-living bacteria reveals a significant overload of tRNA genes in *M. endobia* in relation with its translational requirements (Figure 3). It should be noted that *M. endobia* has multiple tRNAs *loci* for codons that are more frequently represented in *T. princeps* than in itself (Additional files 2 and 3), due to their different G + C content. On the other hand, *T. princeps* has only retained tRNA genes with the anticodon complementary to its most frequently used codons for alanine (GCA) and lysine (AAG). Surprisingly, it has two copies (plus a pseudogene) of the last one, a quite unusual situation for such a reduced genome, while this tRNA is missing in the *M. endobia* genome. This fact might be an indication that *T. princeps* is providing this tRNA to its nested endosymbiont, whose absolute requirements for this tRNA are considerably larger (2032 codons).

Finally, as it was already stated, ribosomes are the best preserved molecular machinery in *T. princeps*[16,19]. In addition to two copies of the ribosomal 23S-16S operon, it encodes 49 out of 56 ribosomal proteins needed to make a complete ribosome. On the other hand, *M. endobia* has also retained a full set of ribosomal proteins and also presents two copies of the 23S and 5S rRNA genes. The high redundancy of rRNA and ribosomal protein genes might indicate that ribosomes from both members of the consortium are not exchangeable, or that redundancy is needed to achieve proper levels of ribosomal components for cell functioning. Both genomes encode the tmRNA, a molecule needed to solve problems that arise during translation while only *M. endobia* encodes ribosome maturation proteins and translational factors.

#### Protein processing, folding and secretion

As compared with their orthologs in free-living relatives, both endosymbionts have retained at least a minimal set of chaperones [35] required for the proper folding of functional proteins in both members of the consortium. This is consistent with the presence of proteins accumulating non-synonymous substitutions. Some proteins can also be exported across the inner and outer membranes via typical gram-negative secretion systems (reviewed in [38]) encoded exclusively in the *M. endobia* genome*.* As other endosymbionts with similarly reduced genomes, *M. endobia* has retained a fully functional Sec translocation complex [16]. It also encodes Ffh, which together with 4.5S RNA forms the signal recognition particle (SRP), needed to bind the signal sequence of the proteins targeted for secretion through this system and to drive them to FtsY, the SRP receptor. Although in other endosymbionts there is an alternative system to assist proteins in their secretion, in which the proteins are recognized by the SecB chaperone after translation, this system cannot be functional in this consortium, because *secB* appears to be a pseudogene [16].

#### Intermediate metabolism

*T. princeps* has almost null metabolic capacities, except for the production of essential amino acids, as described elsewhere [16]. Only *M. endobia* encodes a phosphotransferase system (PTS) for the uptake of hexose as carbon source, and it is predicted to perform glycolysis, transform pyruvate into acetate, and use it to feed the pathway for fatty acids biosynthesis, similarly to that described for *B. aphidicola* BCc, with highly reduced metabolic capabilities [39]. However, the pentose phosphate pathway appears to be incomplete, since only *zwf*, *pgl* and *tkt* have been preserved*,* while *talA* appears to be a pseudogene. Interestingly, *T. princeps* has retained a transaldolase TalB, which along with transketolase (Tkt) creates a reversible link between the pentose phosphate pathway and glycolysis, revealing another possible case of metabolic complementation between both bacteria.

Regarding the tricarboxylic acid (TCA) cycle, only *mdh* (encoding malate dehydrogenase) has been preserved in *T. princeps,* while *M. endobia* has retained only the genes that encode succinyl-CoA synthetase. This is the only step that has been maintained in *S. symbiotica* SCc [5], where the authors indicate that it must have been retained because it is necessary for lysine biosynthesis. Nevertheless, this cannot be the case in this consortium, since lysine biosynthesis cannot be accomplished.

As in other endosymbionts, NAD^+^ can be regenerated by the action of the NADH-quinone oxidoreductase encoded by the *nuo* operon. But, in the absence of ATP synthase coupled to the electron transport chain, the whole consortium relies on substrate-level phosphorylation as a source of ATP. Acetyl-CoA can also be a source of ATP thanks to the presence of the genes *ack*A and *pta*.

The consortium also shares with other endosymbiotic bacteria with reduced genomes the incapability to synthesize nucleotides *de novo*. *T. princeps* has completely lost all genes involved in this function, while *M. endobia* retains a metabolic capacity similar to *B. aphidicola* BCc [39]. All triphosphate nucleotides could be obtained by phosphorylation from diphosphate nucleotides via pyruvate kinase A (*pykA*), while deoxynucleotides could be obtained via ribonucleoside diphosphate reductase 1 (whose subunits are encoded by *nrd*A and *nrd*B). The only preserved diphosphate kinase is adenylate kinase (*adk*), while cytidylate kinase appears to be a pseudogene. Although it has been described that at least one purine and one pyrimidine kinase are needed to phosphorylate all dinucleotides, the fact that Adk is the same kinase that has been preserved in *B. aphidicola* BCc might be an indication that, in endosymbiotic bacteria, this enzyme can act on both nucleotide types. The presence of *dut* guarantees that the thymidylate nucleotides can also be synthesized using dUTP as a primary source.

The endosymbiotic system has almost completely lost the ability to synthesize vitamins and cofactors. Yet, the importance of the [Fe-S] clusters in this consortium is revealed by the presence of complete machinery for the assembly of such components, a complex system that is not fully preserved in other reduced genomes of endosymbiotic bacteria. The [Fe-S] clusters are one of the most ubiquitous and functionally versatile prosthetic groups in nature [40]. Although it is known that these clusters can spontaneously be assembled from the required components under the proper conditions, it is not an efficient procedure *in vivo*[41]. In *E. coli*, their assembly requires a complex machinery and it is achieved by two sets of proteins, the Suf (*suf*ABCDSE) and the Isc (i*sc*SUA) systems. Both members of the consortium are involved in the maintenance of this machinery, revealing another possible case of metabolic complementation. The complete *suf* operon is present in the genome of *M. endobia*. Regarding the Isc system, both partners of the consortium retain *isc*S, and *T. princeps* also encodes *isc*U, while they both lack *isc*A. However, IscA belongs to the HesB family of proteins, and a *hes*B gene has been identified in *T. princeps*. Additionally, ErpA, an A-type iron-sulfur protein that can bind both [2Fe-2S] and [4Fe-4S] clusters, is present in *M. endobia*.

The cell envelope structure is usually highly simplified in Gram-negative endosymbiotic bacteria, which lack most (if not all) of the genes needed for the biosynthesis of murein and lipopolysaccharides, and these two bacteria are not an exception. In fact, *T. princeps* has lost all the genes involved in these functions, and *M. endobia* has also lost many pathways, although it still retains some peptidoglycan synthetases and hydrolases needed for septum formation during cell division. It is noteworthy that this is the first analyzed case of an endosymbiont with a highly reduced genome that retains the ability to synthesize lipid IV_A_, the biosynthetic precursor of lipopolysaccharydes.

#### Cellular transport

Only *M. endobia* has preserved genes related to cellular transport, which must ensure proper exchange of metabolites with the host cell and between both endosymbionts. Many nutrients pass the outer membrane of Gram-negative bacteria via a family of integral outer-membrane proteins (OMPs). The only OMP encoded in the consortium genomes is OmpF, the protein that forms osmotically regulated pores for the passage of small solutes such as sugars, ions and amino acids, with a preference for cationic molecules. Its proper functioning might be essential for the system, since *bam*A (*yae*T) and *bam*D (*yfi*O), coding for the essential components of the assembly machinery of beta-barrel OMPs, as well as *bam*B (*yfg*L), the gene encoding an additional lipoprotein of the system, have been preserved [42]. Additionally, it also retained the two chaperones Skp and SurA, which prevent folding and aggregation of OMPs in the periplasm during passage through the Sec translocon, and assist in their folding once they reach the assembly machinery in the outer membrane, respectively. Although DegP, the protease and chaperone identified to be involved in the degradation of misfolded OMPs, is not present, *M. endobia* encodes DegQ, another periplasmic protease which exhibits functional overlap with its homolog DegP [43,44].

Only a limited set of active transporters are encoded in the *M. endobia* genome. Those include a phosphotransferase system for the transport of hexoses, ABC transporters for zinc, glutathione, lipopolysaccharides and lipidA, as well as a low-affinity inorganic phosphate transporter. Additionally, the *M. endobia* genome also codes for two channels associated with osmotic stress response, MscL and YbaL, which are absent in all Sternorrhyncha endosymbiont genomes sequenced so far. It is worth mentioning that, in addition to low molecular weight molecules, such as ions, metabolites and osmoprotectants, MscL is reported to be involved in the excretion of some small cytoplasmic proteins [45-47]. Therefore, it cannot be ruled out that the preservation of this mechanosensitive channel is an essential part of this peculiar endosymbiont nested system. MscL might be involved in the exchange of molecules between the two bacteria.

## Conclusions

The detailed analysis of the functional capabilities of the two components of the nested endosymbiosis in *P. citri* suggests the existence of an intricate case of complementation, involving not only metabolic but also informational functions. Thus, despite the fact that *M. endobia* resembles *B. aphidicola* BCc [39], another endosymbiont with a highly reduced genome, in many functions such as transport, biosynthesis of cellular envelope and nucleotides, and its incapability to synthesize ATP coupled to the electron transport chain, it possesses particular characteristics that might be related to its coevolution with *T. princeps*. While complementation for amino acid biosynthesis has been described in other endosymbiotic systems, this is the first case in which all energy sources appear to be provided only by one of the partners, similarly to what happens in the eukaryotic cell, where the mitochondria is in charge of this function. Additionally, two genes encoding channels associated with osmotic stress response (*msc*L and *yba*L) have been preserved in its genome. The fact that this kind of molecule has not been identified in other P-endosymbionts with reduced genomes might indicate their connection with special requirements of nested endosymbiosis, and might be involved in the exchange of molecules between both partners.

On the other hand, *T. princeps* does not resemble any known organelle, but it would not be reasonable to consider it, in a strict sense, as a living organism, since it has lost many essential genes involved in informational functions, as well as most metabolic pathways except for the ability to synthesize most essential amino acids, some of which require the cooperation of *M. endobia* and the host [16]. *T. princeps* retains most, but not all, of the translation machinery, for which it also seems to depend on *M. endobia*, even though almost half of its coding capacity is devoted to this function [16,19]. Additionally, it is unable to replicate on its own, although one can hypothesize that composite DNA and RNA polymerases (made of subunits encoded in both genomes) perform this function. *T. princeps* appears to be completely dependent on *M. endobia* for the synthesis of ATP, nucleotides or its cellular envelope, but still retains a complete set of molecular chaperones and proteins needed for the synthesis of [Fe-S] clusters.

Another intriguing fact revealed by our analysis is the overrepresentation of tRNAs genes in the *M. endobia* genome. This fact, together with the duplication in the rRNA operon in both genomes, appears to indicate an important translational activity in which both endosymbionts seem to be engaged. However, it lacks tRNA-Lys(AAG) which, surprisingly, has two functional copies in the small genome of *T. princeps*. This might be an indication that there is a mutual exchange of molecules between both compartments, although further studies are required to demonstrate this.

Nature is prolific in instances of symbiotic cooperation to give rise to new organisms, and new discoveries are always possible. Taking into consideration the deduced exceptional complementation inferred for this endosymbiotic system, we propose that *T. princeps* and *M. endobia* should be considered part of a new composite organism rather than a bacterial consortium.

## Methods

### Insect sample collection and DNA extraction

A population of *P. citri* from an initial sample obtained from a Cactaceae at the Botanical Garden of the Universitat de Valencia (Valencia, Spain) was reared in the laboratory at room temperature, fed on fresh pumpkins and used for genome sequencing. Two other populations of *P. citri* were used for additional experiments. One of them was obtained from a melon field in Murcia (Spain), the second one from a cultured population reared on germinated potatoes at the “Centro de Sanidad Vegetal” (Generalitat Valenciana) in Almassora (Castelló, Spain).

Total DNA enriched in bacterial endosymbionts was extracted from viscera of 20–30 adult female insects in sterile conditions and mechanically homogenized. In order to reduce insect DNA contamination, the samples were subjected to consecutive centrifugations at 1150 g and 1300 g for 10 minutes, and genomic DNA was obtained from the supernatant following a CTAB (Cetyltrimethylammonium bromide) extraction method [48].

### Genome sequencing and assembly

The purified genomic DNA was shotgun sequenced using 454/Roche GS-FLX Titanium technology at the Genomics and Health area of the Public Health Research Center (CSISP, Generalitat Valenciana). One half-plate single-ends, and one-fourth plate paired-ends (3 kb of fragment size) sequencing experiments were performed, yielding a total of 1.3 million reads. Sequences of eukaryotic origin were eliminated after a taxonomic assignation process by Galaxy [49]. Filtered reads were automatically assembled by MIRA [50] and the resulting contigs were manually edited with the Gap4 program from the Staden package software [51]. The remaining gaps in the genome of *M. endobia* str. PCVAL were closed by ABI sequencing of PCR products obtained with designed primers, at the sequencing facility of the Universitat de València. Potential *ori*C on both genomes were sought with the OriginX program [52].

Total DNA samples obtained from the *P. citri* populations from Murcia and Almassora were used to further analyze the *rpl*Q region from the *T. princeps* genome. The region comprised between genes *rpo*A and *aro*K was amplified and sequenced using the primers rpoA-F (5^′^-TGCCAGGCCTAGTGCTAAACATCA-3^′^) and aroK-R (5^′^-TGTCGCCAGGACTGCTATCAATGT-3^′^).

### Gene annotation and functional analysis

ARAGORN [53], tRNAscan [54], and Rfam [55] sowftware packages were used for RNA genes prediction. Coding genes were annotated by BASys (Bacterial Annotation System, [56], RAST [57] and refined by BLAST searches [58]. Finally, functional domain studies in Pfam database [59] were performed when coding-genes functionality assessment was required. Artemis [60] and MEGA5 [61] programs were used for genome statistics calculation and codon usage analysis. Metabolic capabilities were analyzed with Blast2Go [62] and KAAS [63] programs. Functional information from the BioCyc [64], KEEG [65] and BRENDA [66] databases were also used in this context. Genome alignments were performed using MAFFT [67].

Annotated ORFs were considered as functional genes following two non-exclusionary criteria: the conservation of at least 80% of the sequence length of the closest orthologs found by BLAST in non-redundant databases, and/or the maintenance of the essential functional domains detected by Pfam [59].

### Accession numbers

The genome sequence of *M. endobia* strain PCVAL has been deposited at the GenBank (accession number CP003881). The GenBank accession numbers of the other three genome sequences used in this study are as follows: “*Ca.* Tremblaya princeps” str. PCVAL, CP002918; “*Ca.* Tremblaya princeps” str. PCIT, CP002244; *M. endobia* strain PCIT, CP002243.

## Competing interests

The authors declare that they have no competing interests.

## Authors’ contributions

SLM and MP reared and sampled the insects, and performed the DNA extractions. SLM performed the *M. endobia* genome assembly and annotation, and the comparative analyses. SLM and RG performed the functional analysis and prepared figures and tables. RG, AL and AM designed and coordinated the study, and drafted and conducted the manuscript writing. All authors participated on the discussion, reading and approval of the final manuscript.

## Supplementary Material

Additional file 1: Table S1 Differences in gene annotation between strains PCIT and PCVAL for *T. princeps* and *M. endobia*. Gene names refer to the annotation of the PCVAL strain. For those genes duplicated, or encoding hypothetical or unknown proteins, the locus tag is indicated. Gene names or locus tags for the PCIT strain are indicated into brackets when necessary. (+) functional gene; (−) missing gene; (Ψ) pseudogene. Click here for file

Additional file 2: Table S2 Codon usage bias in *T. princeps* PCVAL and *M. endobia* PCVAL. Codon frequencies resulted significantly biased (p-value = 0.01) for all amino acids in *T. princeps*. The same applies to *M. endobia* except for cysteine. In yellow, frequency of the most used codon for the corresponding amino acid in both species. Click here for file

Additional file 3: Table S3 Aminoacyl tRNA synthetases and tRNA genes detected in the *T. princeps* and *M. endobia* genomes. (+) annotated gene; (−) absent gene; (Ψ) pseudogene; (N) number of tRNA isoacceptors detected. Click here for file
