# Supplementary material for: Mealybugs nested endosymbiosis: going into the ‘matryoshka’ system in Planococcus citri in depth
Source: BMC Microbiol. 2013 Apr 1;13:74. doi: 10.1186/1471-2180-13-74 (PMC3620526; doi:10.1186/1471-2180-13-74)
Supplement: Additional file 1: Table S1 — Differences in gene annotation between strains PCIT and PCVAL for T. princeps and M. endobia. Gene names refer to the annotation of the PCVAL strain. For those genes duplicated, or encoding hypothetical or unknown proteins, the locus tag is indicated. Gene names or locus tags for the PCIT strain are indicated into brackets when necessary. (+) functional gene; (−) missing gene; (Ψ) pseudogene. [file 1471-2180-13-74-S1.pdf]

Table S1. Differences in gene annotation between strains PCIT and PCVAL for *T. princeps* and *M. endobia*. Gene names refer to the annotation of the PCVAL strain. For those genes duplicated, or encoding hypothetical or unknown proteins, the locus tag is indicated. Gene names or locus tags for the PCIT strain are indicated into brackets when necessary. (+) functional gene; (-) missing gene; (Ψ) pseudogene.

Species	Gene	Product	Status		Comments
			PCIT	PCVAL	
T. princeps	(*rplU*)	50S ribosomal subunit protein L21	+	-	No significant blastx hits against nr-database
	(*aceF*)	E2 component of pyruvate dehydrogenase	Ψ	-	No significant blastx hits against nr-database
	(TPPCIT_152)	Unknown protein	Ψ	-	No significant blastx hits against nr-database
	(TPPCIT_150)	Hypothetical protein	Ψ	-	No significant blastx hits against nr-database
	(*lpd)*	Lipoamide dehydrogenase	Ψ	-	No significant blastx hits against nr-database
	*leu*A/TCP_127	α-isopropylmalate synthase	-	Ψ	Previously annotated as part of *T. princeps* partial genomic duplication [18]
	*ace*E	Pyruvate dehydrogenase	-	Ψ	Locus identity confirmed by blastx against nr-database
	TCP_012	Hypothetical protein	+	Ψ	One frameshift and an early stop codon with respect to the originally annotated *locus* [18]
	*smp*B	SsrA-binding protein	+	Ψ	Truncated at both extremes, 50% shorter than its closest orthologs. No detection of the corresponding functional domain by Pfam.
	*trp*G	Anthranilate synthase component II	+	Ψ	No detectable similarities against N-terminal extreme of its closest orthologs. Less than 40% of the corresponding functional domain recognized by Pfam.
	TCP_134	Hypothetical protein	+	Ψ	Several frameshifts with respect to the originally annotated *locus* [18]
	tRNA-Lys (TPPCIT_098)	Anticodon=CUU	+	Ψ	Annotated as a pseudogene by tRNAscan-SE
	*grp*E	Heat shock protein	Ψ	+	Retains more than 50% of its closest orthologs; almost 65% of the corresponding functional domain detected by Pfam
	*rpo*D	RNA polymerase, sigma 70 subunit	Ψ	+	Although evidently reduced, 3 (r2, r3 y r4) out of the 5 functional domains in its closest orthologs recognized by Pfam. The lost r1.1 domain has a regulatory role.
	tRNA-Glu	Anticodon=UUC	-	Ψ	Pseudogene predicted by tRNAscan-SE
	*ygb*Q (MEPCIT_471)	Essential cell division protein FtsB	Ψ	+	Shortened, 65% of the corresponding functional domain recognized by Pfam.
M. endobia	MPC_123	Hypothetical protein	Ψ	+	More than 60% gene length retained, showing more than 80% identity with its closest ortholog.
	*ssr*A (MEPCIT_479)	tmRNA (hypothetical protein)	+	+	Different annotation: tmRNA gene identified by Rfam in PCVAL. Hypothetical protein in PCIT.
	*yac*G (MEPCIT_480)	Zinc-binding protein	Ψ	+	*R*etains 70% length of its ortholog in *S. glossinidius*, with 70% identity. The Corresponding functional domain recognized by Pfam
	*ibg*I (MEPCIT_466)	hydrolase-oxidase protein	Ψ	+	More than 72% gene length retained, showing more than 73% identity with its ortholog in *S. glossinidius*. Most of the corresponding functional domain recognized by Pfam
	MPC_265	Hypothetical protein	-	Ψ	Annotation based on blastx and synteny analysis, compared with *S. glossinidius* (SG0460)
	*pdx*J/MPC_306	Pyridoxine 5'-phosphate synthase	-	Ψ	Pseudogene involved in the partial genomic duplication
