# Supplementary material for: Mealybugs nested endosymbiosis: going into the ‘matryoshka’ system in Planococcus citri in depth
Source: BMC Microbiol. 2013 Apr 1;13:74. doi: 10.1186/1471-2180-13-74 (PMC3620526; doi:10.1186/1471-2180-13-74)
Supplement: Additional file 2: Table S2 — Codon usage bias in T. princeps PCVAL and M. endobia PCVAL. Codon frequencies resulted significantly biased (p-value = 0.01) for all amino acids in T. princeps. The same applies to M. endobia except for cysteine. In yellow, frequency of the most used codon for the corresponding amino acid in both species. [file 1471-2180-13-74-S2.pdf]

Table S2. Codon usage bias in *T. princeps* PCVAL and *M. endobia* PCVAL. Codons frequencies resulted significantly biased (p-value=0.01) for all amino acids in *T. princeps*. The same applies to *M. endobia* except for cysteine. In yellow, frequency of the most used codon for the corresponding amino acid in both species.

		T. princeps		***M. endobia***	
**AA**	**Codons**	**Abundance**	**%**	**Abundance**	**%**
Pro	CCA	454	36.09	1953	37.08
	CCT	291	23.13	1245	23.64
	CCG	274	21.78	1514	28.75
	CCC	239	18.99	555	10.54
Arg	AGG	1340	42.27	372	4.53
	CGC	747	23.57	2677	32.62
	CGG	331	10.44	818	9.96
	CGT	289	9.11	3005	36.60
	AGA	253	7.98	552	6.72
	CGA	210	6.63	785	9.56
Ala	GCA	1139	31.15	3255	27.95
	GCC	897	24.53	2505	21.49
	GCG	857	23.44	2349	20.16
	GCT	764	20.89	3543	30.40
Gly	GGC	1316	48.63	3107	32.35
	GGG	526	19.44	898	9.35
	GGA	450	16.63	1473	15.34
	GGT	414	15.30	4125	42.96
Leu	CTG	907	33.30	3364	22.85
	CTA	613	22.50	3094	21.01
	CTT	482	17.70	2070	14.06
	CTC	459	16.85	659	4.47
	TTG	172	6.31	2720	18.47
	TTA	91	3.34	2818	19.15
Val	GTG	895	35.76	2503	25.52
	GTA	595	23.77	3005	30.63
	GTT	563	22.49	2987	30.46
	GTC	450	17.98	1313	13.39
His	CAC	604	62.01	1103	32.12
	CAT	370	37.99	2330	67.88
Gln	CAG	610	81.33	3320	54.15
	CAA	140	18.67	2808	45.85
Asp	GAC	770	62.00	2027	29.63
	GAT	472	38.00	4815	70.37
Glu	GAG	933	81.20	2217	28.77
	GAA	216	18.80	5490	71.23
Ser	AGC	686	30.71	1753	22.28
	TCG	393	17.60	913	11.61
	TCA	384	17.12	1297	16.49
	TCC	366	16.38	910	11.57
	TCT	286	12.80	1451	18.47
	AGT	119	5.33	1541	19.59
Thr	ACG	434	30.80	1026	13.52
	ACC	366	25.98	2501	32.96
	ACA	340	24.13	1578	20.83
	ACT	269	19.10	2481	32.69
Cys	TGC	747	84.31	896	50.20
	TGT	139	15.69	889	49.80
Trp	TGG	235	100	1469	100
Ile	ATA	711	53.10	2444	24.33
	ATC	354	26.44	2781	27.70
	ATT	274	20.46	4818	47.97
Met	ATG	981	100	3793	100
Asn	AAC	473	74.96	2542	40.70
	AAT	158	25.04	3702	59.30
Lys	AAG	602	83.50	2032	29.10
	AAA	119	16.50	4951	70.90
Phe	TTC	485	81.93	1699	34.99
	TTT	107	18.07	3154	65.01
Tyr	TAC	499	72.11	1444	33.97
	TAT	193	27.89	2805	66.03
