# Supplementary material for: Mealybugs nested endosymbiosis: going into the ‘matryoshka’ system in Planococcus citri in depth
Source: BMC Microbiol. 2013 Apr 1;13:74. doi: 10.1186/1471-2180-13-74 (PMC3620526; doi:10.1186/1471-2180-13-74)
Supplement: Additional file 3: Table S3 — Aminoacyl tRNA synthetases and tRNA genes detected in the T. princeps and M. endobia genomes. (+) annotated gene; (−) absent gene; (Ψ) pseudogene; (N) number of tRNA isoacceptors detected. [file 1471-2180-13-74-S3.pdf]

Table S3. Aminoacyl tRNA synthetases and tRNA genes detected in the *T. princeps* and *M. endobia* genomes. (+) annotated gene; (-) absent gene; (Ψ) pseudogene; (N) number of tRNA isoacceptors detected.

	T. princeps			*M. endobia*		
tRNA	Anticodons	N	Aminoacyl tRNA synthetase	Anticodons	N	Aminoacyl tRNA synthetase
tRNA-Ala	UGC	1	-	GGC, UGC	2	+
tRNA-Gly	-	-	-	GCC, UCC	2	+
tRNA-Pro	-	-	-	UGG, CGG, GGG	3	+
tRNA-Thr	-	-	-	GGU, UGU, CGU	3	+
tRNA-Val	-	-	-	UAC, GAC	2	+
tRNA-Ser	UGA	1	-	GGA, CGA, UGA, GCU	4	+
tRNA-Arg	ACG	1	Ψ	ACG, CCG, CCU, UCU	3	+
tRNA-Leu	-	-	-	UAG, CAG, GAG, UAA, CAA	5	+
tRNA-Phe	GAA	Ψ	-	GAA	1	+
tRNA-Asn	-	-	-	GUU	1	+
tRNA-Lys	CUU	2, Ψ	-	UUU	1	+
tRNA-Asp	-	-	-	GUC	2	+
tRNA-Glu	UUC	Ψ	-	UUC	1	+
tRNA-His	-	-	-	GUG	1	+
tRNA-Gln	-	-	-	UUG, CUG	2	+
tRNA-Ile	GAU	Ψ	-	GAU	1	+
tRNA-Met	CAU	1	-	CAU	3	+
tRNA-Tyr	-		-	GUA	1	+
tRNA-Cys	-		Ψ	GCA	1	+
tRNA-Trp	CCA	1	-	CCA	1	+
